# Neurological symptoms and comorbidity profile of hospitalized patients with COVID-19

**DOI:** 10.1055/s-0043-1761433

**Published:** 2023-03-22

**Authors:** Renata Carvalho Cremaschi, Carla Alessandra Scorza Bahi, Angelo Amato Vincenzo de Paola, Jaquelina Sonoe Ota Arakaki, Paulo Roberto Abrão Ferreira, Nancy Cristina Junqueira Bellei, Vanderci Borges, Fernando Morgadinho Santos Coelho

**Affiliations:** 1 Universidade Federal de São Paulo, Departamento de Neurologia e Neurocirurgia, São Paulo SP, Brazil.; 2 Universidade Federal de São Paulo, Departamento de Medicina, São Paulo SP, Brazil.; 3 Universidade Federal de São Paulo, Departamento de Psicobiologia, São Paulo SP, Brazil.

**Keywords:** COVID-19, Neurologic Manifestations, Comorbidity, Anosmia, Orthostatic Intolerance, Autonomic Nervous System Diseases, COVID-19, Manifestações Neurológicas, Comorbidade, Anosmia, Intolerância Ortostática, Doenças do Sistema Nervoso Autônomo

## Abstract

**Background** The neurological manifestations in COVID-19 adversely impact acute illness and post-disease quality of life. Limited data exist regarding the association of neurological symptoms and comorbid individuals.

**Objective** To assess neurological symptoms in hospitalized patients with acute COVID-19 and multicomorbidities.

**Methods** Between June 2020 and July 2020, inpatients aged 18 or older, with laboratory-confirmed COVID-19, admitted to the Hospital São Paulo (Federal University of São Paulo), a tertiary referral center for high complexity cases, were questioned about neurological symptoms. The Composite Autonomic Symptom Score 31 (COMPASS-31) questionnaire was used. The data were analyzed as a whole and whether subjective olfactory dysfunction was present or not.

**Results** The mean age of the sample was 55 ± 15.12 years, and 58 patients were male. The neurological symptoms were mostly xerostomia (71%), ageusia/hypogeusia (50%), orthostatic intolerance (49%), anosmia/hyposmia (44%), myalgia (31%), dizziness (24%), xerophthalmia (20%), impaired consciousness (18%), and headache (16%). Furthermore, 91% of the patients had a premorbidity. The 44 patients with subjective olfactory dysfunction were more likely to have hypertension, diabetes, weakness, shortness of breath, ageusia/hypogeusia, dizziness, orthostatic intolerance, and xerophthalmia. The COMPASS-31 score was higher than that of previously published controls (14.85 ± 12.06 vs. 8.9 ± 8.7). The frequency of orthostatic intolerance was 49% in sample and 63.6% in those with subjective olfactory dysfunction (2.9-fold higher risk compared to those without).

**Conclusion** A total of 80% of inpatients with multimorbidity and acute COVID-19 had neurological symptoms. Chemical sense and autonomic symptoms stood out. Orthostatic intolerance occurred in around two-thirds of the patients with anosmia/hyposmia. Hypertension and diabetes were common, mainly in those with anosmia/hyposmia.

## Resumo

**Antecedentes** As manifestações neurológicas na COVID-19 impactam adversamente na enfermidade aguda e na qualidade de vida após a doença. Dados limitados existem em relação a associação de sintomas neurológicos e indivíduos com comorbidades.

**Objetivo** Avaliar os sintomas neurológicos em pacientes de hospitalizados com COVID-19 aguda e múltiplas comorbidades.

**Métodos** Entre junho e julho de 2020, pacientes de hospitais com idade 18 anos ou acima e COVID-19 laboratorialmente confirmada, admitidos no Hospital São Paulo (Universidade Federal de São Paulo), um centro de referência terciário para casos de alta complexidade, foram perguntados sobre sintomas neurológicos. O questionário Pontuação composta de sintoma autonômico (COMPASS-31) foi usado. Os dados foram analisados no geral e se a disfunção olfatória subjetiva estava presente ou não.

**Resultados** A média de idade da amostra foi 55 ± 15.12 anos. 58 pacientes eram homens. Os sintomas neurológicos foram principalmente xerostomia (71%), ageusia/hipogeusia (50%), intolerância ortostática (49%), anosmia/hiposmia (44%), mialgia (31%), tontura (24%), xeroftalmia (20%), comprometimento na consciência (18%) e cefaleia (16%). Além disso, 91% dos pacientes tinham uma pré-morbidade. Os 44 pacientes com disfunção olfatória tinham maior chance de ter hipertensão, diabetes, fraqueza, falta de ar, ageusia/hipogeusia, tontura, intolerância ortostática e xeroftalmia. A pontuação do COMPASS-31 foi maior do que a de controles previamente publicados (14,85 ± 12,06 vs. 8,9 ± 8,7). A frequência de intolerância ortostática foi 49% na amostra e 63,6% naqueles com disfunção olfatória subjetiva (risco 2.9 vezes maior comparado com os sem).

**Conclusão** Um total de 80% dos pacientes hospitalizados com múltiplas morbidades e COVID-19 aguda tinham sintomas neurológicos. Os sintomas do sentido químico e autonômicos se destacaram. A intolerância ortostática ocorreu em cerca de dois terços dos pacientes com anosmia/hiposmia. A hipertensão e o diabetes foram comuns, principalmente naqueles com anosmia/hiposmia.

## INTRODUCTION

The novel severe acute respiratory syndrome coronavirus 2 (SARS-CoV-2) binds mainly to the angiotensin-converting enzyme type 2 (ACE2) receptor in the olfactory and respiratory epithelium. The virus's interaction can cause anosmia/hyposmia. Because ACE2-expressing cells are widely distributed, the SARS-CoV-2 reaches extrapulmonary sites as well. 1

The neurological manifestations were early linked to the coronavirus disease 2019 (COVID-19). 2 The pathophysiology is related to immune activation, neuroinflammation, and damage to brains blood vessels. 3 Anosmia was associated with low in-hospital mortality. 4 5 Although chemosensory symptoms are primary presentations of neurological features in COVID-19, a broad spectrum of other neurological symptoms accompany them. A sizeable proportion of the patients with anosmia have headache, and others develop mild-to-severe complications, including life-threatening cerebrovascular events. 6 7

A few symptoms of the illness overlap with the symptoms of autonomic dysfunction. Particularly in the early stages of COVID-19, syncope and silent hypoxemia are the clinical presentations of autonomic impairment, which could be associated with the intrinsic mechanism of the viral infection or other non-COVID causes. 8 Some patients in recovery have experienced long-term autonomic symptoms, which are reported in long-COVID. 9 10 Cardiovascular autonomic dysfunction was confirmed. 11 Dysautonomia was overlooked in critically ill COVID-19 patients. 12

The studies evaluating COVID-related neurological manifestations are heterogeneous, because they include symptoms and complications. The association between high-risk preexisting comorbidities for COVID-19 hospitalization and related deaths has been established in the literature. 13 About 43.9% of the patients with COVID-19 and neurological symptoms have comorbid conditions. 14 The impact of morbidities in the neurological symptoms of COVID-19, especially those with a strong ACE2 receptor expression that enhances the viral entry into the host cells, has not been well emphasized.

Even though anosmia/hyposmia is one of the most frequent neurological symptoms observed, not much is known regarding the differences of the other neurological symptoms and the preexisting diseases in patients, regardless of whether subjective olfactory impairment is present or not. This study investigates inpatients with multiple comorbidities from a public reference hospital of Sao Paulo, in the course of the initial outbreak of the pandemic. The objectives were to assess the frequency of neurological symptoms in patients hospitalized for the treatment of COVID-19. We also evaluated the association between subjective anosmia/hyposmia and other neurological symptoms or comorbidities.

## METHODS

### Standard protocol approval and patient consent

The University's Institutional Ethics Committee (Comitê de Ética em Pesquisa da Universidade Federal de São Paulo) approved the study, with the ethics board approval number 0547/2020. All participants in the study provided written informed consent for the research.

### Study design and sample

This cross-sectional study describes neurological symptoms in patients admitted to a public tertiary referral center for high complexity cases, which turned into a COVID-19 care center. The study was conducted at the São Paulo Hospital, from the Federal University of Sao Paulo (Brazil).

The inclusion criteria were symptomatic patients aged 18 or older, with diagnostic confirmation of SARS-CoV-2 infection by nasopharyngeal swab reverse-transcriptase polymerase chain reaction (RT-PCR). The exclusion criteria were pregnancy or puerperium, dementia as a premorbidity, assessment during mechanical ventilation, or hemodynamic instability. The sampling method was noncasual and convenient, intended to represent a sample of real-life inpatients.

### Data acquisition

The hospital capacity was organized to treat COVID-19 patients separately. The occupancy rates were high, but an exact overall value was inaccurate. All patients sited at the infectious diseases and pneumology units (48 beds available) were invited to participate, between June 2020 and July 2020. Data collection was performed once a week, during five consecutive weeks. Before recruitment, the assistant physician confirmed the stable clinical condition of each patient, enabling them to join in the research. Two expert neurologists (FMSC, RMCC) interviewed the patients to fill out electronic forms with answers on the experienced symptoms. The completion of all items was checked before sending them for data processing. Clinical data were retrieved manually from electronic medical charts. The reported medications comprised those in use before admission.

According to the manufacturer's protocols, all quantitative RT-PCR analyses were made in a single laboratory located at the hospital. Additionally, RNA was isolated from the subject's nasopharyngeal swabs using the Quick-RNA Viral Kit (Zymo Research, Irvine, CA, USA). After extraction, the samples were tested for SARS-CoV-2 using a multiplex RT-PCR commercial kit (Gene Finder, South Korea). Early in the first wave of COVID-19, the identification of the variants was not yet implemented. Different commercially available kits were used in the samples of subjects who had been initially diagnosed at other hospitals. At admission, the degree of radiological involvement was confirmed using chest computed tomography to identify the typical ground-glass opacities extension.

### Main outcome measure

#### Neurological symptoms

As there was no standardized measure of neurological symptoms in COVID-19, a survey was created, based on those of the initial report in China. 2 The Composite Autonomic Symptom Score 31 (COMPASS-31) was added to investigate symptoms of orthostatic intolerance and secretomotor function, which are frequently underrecognized. Later, in the context of long-COVID, this questionary was suggested as a screening tool to track symptoms changes of autonomic dysfunction. 15

Olfactory dysfunction (OD), partial hyposmia or total anosmia, is the reduced ability to smell during sniffing (orthonasal olfaction) or eating and drinking (retronasal olfaction). The retronasal olfaction contributes to the taste, whose dysfunction can be partial hypogeusia or total ageusia. The interviewers asked whether smell and taste were reduced or absent during sniffing, chewing, drinking, or digesting food. Orthostatic intolerance, xerophthalmia, and xerostomia were identified according to questions number one, nine, and ten of COMPASS-31. Questions number two and three characterized the frequency and intensity of orthostatic intolerance.

The COMPASS-31 is a 31-item survey, organized in 6-scale domains, which assesses the presence, severity, distribution, frequency, and progression of autonomic symptoms. Regarding the clinical relevance of the domain, the scores provide the total weighted score, which ranges from 0 to 100. The higher scores indicate a more significant symptom load. 16 As some COVID-19 symptoms are similar to autonomic symptoms, a score with gastrointestinal domain scored as zero was named COMPASS-31(-GI). The Mayo Clinic Autonomic Laboratory validated the Brazilian Portuguese language version of COMPASS-31. The overall score of patients with COVID-19 was compared to those of healthy controls in previously published literature (mean 8.9 ± 8.7). 17

### Comorbidity profile

As a complete database was available, the co-existing conditions were obtained manually in medical records. The Charlson Comorbidity Index was calculated by the investigators. The method uses 17 comorbidities associated with mortality to classify prognostic comorbidity. 18 The severity of comorbid diseases is mild (scores 1–2), moderate (scores 3–4), and severe (scores ≥ 5).

### Statistical analysis

Data were examined using the Statistical Package Social Sciences (SPSS, IBM Corp. Armonk, NY, USA) software for windows 8, version 21.0. Data were analyzed as a whole, and whether the patient has subjective OD or not (s̅OD). Normality and homogeneity tests and parametric analyses were used in the descriptive statistics. The Student T-test was used to compare COMPASS-31 overall total weighted score (TWS) and controls. The Pearson chi-squared and Fisher exact tests were used to analyze categorical variables. Generalized Linear Models, according to the distribution of the dependent variable (gamma and Poisson log-linear), were used to obtain COMPASS-31 scores, with OD as the independent variable. Logistic binary regression, with OD as the predictor, was used to compare the magnitude of the risk factors (odds ratio, OR) in a few neurological symptoms. The results are presented as means ± standard deviation and percentages (%). The significant level considered was *p* < 0.05.

## RESULTS

### Demographics

A total of 124 surveys were obtained among the 118 patients enrolled. After reviewing the medical records, our study consisted of 100 patients. The clinical, laboratory, and imaging features of the patients are summarized in Table 1 . Among the sample, 44 patients had OD. The mean age of the sample was 55.02 ± 15.12 years, with 39 patients being over 60 years of age. There were 58 male patients. The sample's mean body mass index (BMI) was above normal limits. Furthermore, OD patients had a higher percentage of weakness, shortness of breath, and constipation, compared to s̅OD. The mean lymphocyte count was also higher for OD patients than for s̅OD. There was a higher percentage of thrombocytopenia in s̅OD, compared with OD. Regarding chest imaging, 60.7% of the patients in the s̅OD group had < 25% disease severity, and 43.2% of those in the OD group had 25 to 49% extension.

**Table 1:** Clinical, laboratory, and imaging features of hospitalized patients with COVID-19

		Overall	OD		*p-* value
			without	with	
		(N = 100)	(N = 56)	(N = 44)	
Age, years		55.02 ± 15.12	55.16 ± 15.26	54.84 ± 15.11	0.91
Age category, %	18–60 years	61	57.1	65.9	0.37
	>60 years	39	42.9	34.1	
Male gender, %		58	66.1	47.7	0.06
BMI, kg/m ^2^		27.66 ± 5.96	27.49 ± 5.38	27.79 ± 6.42	0.80
Regular medications use	Antihypertensives ^1^	66	67.9	63.6	0.65
	Antidepressants and neuroleptics	6	3.6	9.1	0.40
	Immunosuppressants	31	30.4	31.8	0.87
	Oral corticosteroids	27	26.8	27.3	0.95
	Oral anticoagulants	4	3.6	4.5	1
**General symptoms, %**	Anorexia	50	42.9	59.1	0.10
	Fever/chill	66	66.1	65.9	0.98
	Weakness	60	50.0	72.7	**0.02**
	Cough	68	62.5	75.0	0.18
	Sore throat	25	19.6	31.8	0.16
	Rhinorrhea	21	23.2	18.2	0.54
	Shortness of breath	66	57.1	77.3	**0.03**
	Diarrhea	50	50	50	1
	Nausea/vomiting	35	35.7	34.1	0.86
	Constipation	15	7.1	25	**0.01**
Viral load quantitation, %	High	44	40.0	59.5	0.07
	Low	45	60.0	40.5	
	Missing ^**2**^	13	6	7	
**Delay to confirmation, days** ^3^		6.08 ± 4.24	6.25 ± 4.49	5.86 ± 3.93	0.65
** White blood cells ^4^**	Leucocyte count	9,164 ± 6,581	8,636 ± 6,831	9,835 ± 6,262	0.36
	Neutrophils count	6,859 ± 4,815	6,638 ± 5,286	7,140 ± 4,182	0.60
	Lymphocytes count	1,202 ± 876	1,011 ± 724	1,445 ± 995	**0.01**
**Platelet**	Count ^**4**^ *1000	214.85 ± 95.71	198.84 ± 92.86	235.23 ± 96.45	0.05
	Thrombocytopenia, % (<100,000 ^**4**^ )	27	35.7	15.9	**0.02**
** Serum creatinine ^5^**		2.82 ± 7.07	2.28 ± 3.04	3.51 ± 10.12	0.39
** Blood urea nitrogen ^5^**		63.47 ± 53.59	66.29 ± 61.09	59.95 ± 42.85	0.56
** C-reactive protein ^6^**		104.23 ± 79.82	104.96 ± 78.76	103.3 ± 82.07	0.91
** Lactose dehydrogenase ^7^**		384.12 ± 164.71	351.3 ± 140.63	420.23 ± 182.7	0.05
** Chest CT category, % ^8^**	0 (none)	7	5.4	9.1	**0.001**
	1 (<25%)	43	60.7	20.5*	
	2 (25–49%)	31	21.4	43.2*	
	3 (50–75%)	18	12.5	25.7	
	4 (>75%)	1	0.0	2.3	

**Abbreviations:** BMI, body mass index; RT-PCR, reverse-transcriptase polymerase chain reaction; CT, computed tomography; OD, olfactory dysfunction. **Notes:** Values are expressed as the mean ± standard deviation or percentage (%). Bold numbers indicate statistical significance. * *p* -value < 0.05 from analysis of variance and the Pearson chi-squared or Fisher exact tests within the groups without and with OD. Olfactory dysfunction = subjective anosmia/hyposmia. ^1^ Angiotensin-converting-enzyme inhibitors, diuretics, and vasodilators. ^2^ Samples collected at another hospital. ^3^ From symptom onset to RT-PCR. ^4^ per mm3. ^5^ mg/dL. ^**6**^ mg/liter. ^7^ U/liter. ^8^ Multifocal ground-glass opacities and consolidation as disease severity.

Both groups had a high percentage of patients on antihypertensive agents, and a low percentage on antidepressants and neuroleptics. Almost all the patients were using ACE inhibitors. The viral load quantitation was missing for 13 patients who had the samples collected at a different hospital. There were no significant differences between the groups in the use of medications, other general symptoms, delay to confirm SARS-Cov2 infection, and other laboratory testing on admission. The Supplementary Material Table S1 shows in-hospital standard of care of the patients. Finally, the OD group received more antiviral treatment than s̅OD (11.4 vs. 1.8%), but other differences were not seen, including clinical outcomes and mortality.

### Neurological symptoms

Table 2 shows that the mean number of days from the onset of illness to the neurological symptoms was 1.48 ± 3.41. The neurological symptoms occurred in 80% of the sample. The patients in the s̅OD group reported 64.3% of other neurological symptoms rather than anosmia/hyposmia. The most frequent symptoms were xerostomia (71%), ageusia/hypogeusia (50%), orthostatic intolerance (49%), anosmia/hyposmia (44%), myalgia (31%), dizziness (24%), xerophthalmia (20%), impaired consciousness due to syncope, seizures, daytime sleepiness or insomnia (18%), and headache (16%). The OD group had a higher percentage of ageusia/hypogeusia (97.7 vs. 12.5%, OR = 301, 95% confidence interval [CI] 35.59 to 2,545.42), dizziness (34.1 vs. 16.1%, OR = 2.70, 95% CI 1.04 to 6.96) orthostatic intolerance (63.6 vs. 37.5%, OR = 2.91, 95% CI 1.28 to 6.61), and xerophthalmia (29.5 vs. 12.5%, OR = 2.93, 95% CI 1.05 to 8.16) than s̅OD. Orthostatic intolerance occurred more frequently and with moderate and severe intensity in OD. Other neurological symptoms did not differ. The sample had a higher COMPASS-31 mean score than that of healthy controls previously published (14.85 ± 12.06 vs. 8.9, t = 4.939, 95% CI 3.56 to 8.35, *p* < 0.001). The COMPASS-31 and COMPASS-31(-GI) mean scores for the OD group were higher than for the s̅OD one.

**Table 2:** Neurological symptoms of hospitalized patients with COVID-19

		OD		
	Overall	without	with	p *-* value
	(N = 100)	(N = 56)	(N = 44)	
** Delay, days ^1^**	1.48 ± 3.41	1.39 ± 3.48	1.55 ± 3.39	0.84
**Neurological symptom, %**	80	64.3	100	**<0.001**
Ageusia/hypogeusia ^2^	50	12.5	97.7	**<0.001**
Vision impairment	11	8.9	13.6	0.45
Dizziness ^3^	24	16.1	34.1	**0.03**
Irritability	12	7.1	18.2	0.09
Impaired consciousness ^4^	18	23.2	11.4	0.12
Confusion	9	8.9	9.1	0.97
Muscle weakness ^5^	2	1.8	2.3	1
Myalgia	31	23.2	40.9	0.05
Headache	16	12.5	20.5	0.28
Orthostatic intolerance ^6^	49	37.5	63.6	**0.01**
*Frequency*				
Never	51	62.5	36.4*	**0.02**
Rarely	14	12.5	15.9	
Occasionally	19	17.9	20.5	
Frequently	15	7.1	25*	
Almost always	1	0	2.3	
*Intensity*				**0.01**
None	51	62.5*	36.4*	
Mild	23	23.2	22.7	
Moderate	20	12.5	29.5*	
Severe	6	1.8*	11.4*	**0.03**
Xerophthalmia ^7^	20	12.5	29.5	**0.03**
Xerostomia	71	64.3	79.5	0.09
**COMPASS-31 TWS** (0–100)	16.69 ± 11.52	12.92 ± 13.72	20.90 ± 23.47	**0.002**
**TWS minus GI** (0–75)	13.82 ± 9.71	10.58 ± 12.16	17.21 ± 20.26	**0.003**

**Abbreviations:** COMPASS-31, Composite Autonomic Symptom Score 31; TWS, total weighted score; () or parenthesis, maximum possible score; GI, gastrointestinal; CI, confidence interval; OD, olfactory disfunction. **Notes:** Values expressed as the mean ± standard deviation or percentage (%). Bold numbers indicate statistical significance. * *p-* value < 0.05. Categorical variables analysis performed by the Pearson chi-squared or Fisher exact tests. Continuous variables and odds ratio analysis performed by generalized linear model within the groups without and with OD. Olfactory dysfunction = subjective anosmia/hyposmia. ^1^ Onset of illness to manifestation. ^2^ OR = 301, 95%CI 35.59 to 2,545.42. ^3^ OR = 2.70, 95%CI 1.04 to 6.96. ^4^ Syncope, seizure, daytime sleepiness, insomnia. ^5^ Due to acute cerebrovascular disease. ^6^ OR = 2.91, 95%CI 1.28 to 6.61. ^7^ OR = 2.93, 95%CI 1.05 to 8.16.

### Comorbidity profile

The associated diseases are presented in Table 3 . The majority of the patients (91%) had a comorbid condition. The most frequent premorbidities were hypertension (60%), diabetes (40%), obesity (33%), smoking history (25%), and cardiac (23%) or end-stage renal (31%) diseases. The OD group had a higher percentage of hypertension (72.7 vs. 50%) and diabetes (59.1 vs. 25%), compared to s̅OD. There were no significant differences between these groups concerning other coexisting disorders or the Charlson Comorbidity Index, according to which 65% of the patients had a moderate to severe scores.

**Table 3:** Comorbidity profile of hospitalized patients with COVID-19

		OD		
	Overall	without	with	*p-* value
	(N = 100)	(N = 56)	(N = 44)	
**Associated medical disease, %**				
Hypertension	60	50	72.7	**0.02**
Diabetes mellitus	40	25	59.1	**0.001**
Obesity (BMI > 30 kg/m ^2^ )	33	32.1	34.1	0.83
Smoking history ^1^	25	26.8	22.7	0.64
Cardiac diseases ^2^	23	25	20.5	0.59
Chronic pulmonary disease ^3^	10	12.5	6.8	0.34
End-stage renal disease	31	30.4	31.8	0.87
Kidney transplantation	24	23.2	25	0.83
Hemodialysis	7	7.1	6.8	1
Malignancy	9	8.9	9.1	0.97
Asthma	7	3.6	11.4	0.23
Vasculiti ^4^	7	8.9	4.5	0.46
Cerebrovascular	7	3.6	11.4	0.23
Peripheral artery and aneurysm	2	0	4.5	0.37
Falciform anemia	2	1.8	2.3	1
Pulmonary tuberculosis	1	1.8	0	1
Other ^5^	24	30.4	15.9	0.09
**Charlson Comorbidity Index grade, %**				0.64
*None (CCI = 0)*	9	10.7	6.8	0.49
*Mild (CCI = 1* – *2)*	26	26.8	25	0.84
*Moderate (CCI = 3* – *4)*	39	41.1	36.4	0.63
*Severe (CCI ≥ 5)*	26	21.4	31.8	0.24

**Abbreviations:** BMI, body mass index; CCI, Charlson Comorbidity Index; OD, olfactory disfunction. **Notes:** Values expressed as the mean ± standard deviation or percentage (%). Bold numbers indicate statistical significance. * *p-* value < 0.05 from analysis of variance and the Pearson chi-squared or Fisher exact tests within the groups without and with OD. Olfactory dysfunction = subjective anosmia/hyposmia. ^1^ Former and current. ^2^ Coronary heart disease, chronic chagasic cardiopathy, arrhythmia, congestive heart failure. ^3^ Obstructive and restrictive. ^4^ Sarcoidosis, systemic sclerosis, systemic sclerosis, and lupus. ^5^ Infectious diseases (HIV, hepatitis, syphilis, schistosomiasis); cerebral palsy; Parkinson disease; cocaine addiction; alcohol abuse; chronic pancreatitis; and other comorbidities.

## DISCUSSION

Among hospitalized multimorbidity patients with laboratory-confirmed COVID-19, the neurological symptoms were very frequent, developing shortly after the onset of typical flu symptoms. Our strong results are the presence of chemical sense impairment and underestimated autonomic symptoms. Most of the patients without anosmia/hyposmia also reported another neurological symptom.

The neurological features in COVID-19 vary according to the sample and data collection. Our frequency of olfactory and gustatory dysfunction is comparable to the 46.8 and 52.3% already described. Headache and dizziness were reported in 7.5% and 6.1% of patients, respectively. Diabetes (31.1%) and hypertension (13.5%) were the most common associated comorbidities . 19 The pooled prevalence of central nervous system or mental associated disorders in COVID-19 is around 50.68%. The most frequent symptom was OD (36.20% in 10 studies of the meta-analysis). 20 The main neurological symptoms detected among COVID-19 patients were fatigue (42.9%), gustatory (35.4%) and olfactory (25.3%) dysfunctions, 10% headache (10%), and dizziness (6.7%) in another meta-analysis. 14 An European multinational study with 6,537 SARS-infected patients reported the frequency of headache (18.5%), impaired sense of smell (9.0%) and taste (12.8%), and delirium (6.7%). The patients were mostly hospitalized in complicated/critical (53%) and uncomplicated phases. 21

As expected, the three most frequent diseases found in our patients are risk factors for COVID-19, which seem to negatively affect the clinical course and morbimortality outcomes, but this is not definitely clear. 22 Our groups without and with OD were demographically similar, but the one with OD constituted a majority of those with hypertension, diabetes, and a higher extension on chest imaging.

The SARs-CoV-2 upregulates ACE2 expression in patients with hypertension, which can increase blood pressure and determine pneumonia. As diabetes has impaired T-cell function and increased interleukin-6, pneumonia-like symptoms can exist in these patients with COVID-19. 23 The association between COVID-19 and hypertension has generated considerable discussion. Hypertension is normally accompanied by many comorbidities that are major factors for disease severity, and there is little evidence of ACE inhibitors influencing tissue expression or activity of ACE2 in humans. 24

The increased risk of hyposmia after recovery of patients with type 2 diabetes and mild pneumonia associated with COVID-19, as well as animal experiments, indicate that diabetes could dampen the first-line defense of nasal immunity. There is an impaired nasal-associated lymphoid tissue immunity in diabetes type 2. 25 The extended autonomic system includes the neuroendocrine and neuroimmune systems. 18 The link between diabetes and OD is controversial. Adults with diabetes on more aggressive treatments showed a trend toward severe hyposmia/anosmia in the pocket smell test, without an association between disease duration and self-reported symptoms. 26 There were no differences in an objective odor test between patients with and without chronic complications of diabetes. 27

The slightly increased autonomic load in our sample might be explained, at least partially, by diabetes. The COMPASS-31 scores in all the groups of this study were lower than those of diabetics with cardiovascular autonomic neuropathy and polyneuropathy. 28 A cut-off of 28.67 indicates autonomic dysfunction in diabetes. 29 Even though the symptoms were interrogated during COVID-19, and despite the confounders, such as medication side effects and dehydration, diabetes was a possible factor for orthostatic intolerance in our study.

The elevated frequency of ageusia/hypogeusia in those with OD in our study was likely due to gustatory function being mediated through the sense of smell, pain perception, and somatosensory pathway. Elevated olfactory and gustatory dysfunction occur in mild-to-moderate COVID-19 through sense questionnaires. Of the 18.2% of patients without nasal obstruction or rhinorrhea, 79.7% had OD. 7 Chemosensory loss of smell and taste occurred in outpatients with influenza-like symptoms and COVID-19. 30

Our frequency of headache was a quarter of the one detected in adult symptomatic patients with laboratory-confirmed COVID-19 from the northeast of Brazil, whose characteristics were usually bilateral and severe, but rarely continuous. We found a comparable frequency of anosmia/hyposmia and ageusia/hypogeusia, but not the elevated risk of headache in those with impairment of smell and taste. 6

The autonomic symptoms can arise as para- and postinfectious manifestations, especially in viral infections such as herpes simplex and mononucleosis. A case-control study from Colombia showed that in Zika virus outbreaks, the COMPASS-31 was elevated, mainly in the orthostatic, secretomotor, and bladder domains, about 63 weeks after disease onset. 31

Two-thirds of the patients with anosmia/hyposmia had orthostatic intolerance answering to: *você se sentiu tonto, desorientado, aéreo ou teve dificuldade de pensar quando levantou após ter ficado sentado ou deitado?* (did you feel dizzy, disoriented, lightheaded or had difficulty thinking when you got up from sitting or lying down?). They had a 2.9-fold higher risk, compared to those without anosmia/hyposmia. Orthostatic intolerance was more frequent and more intense in those with anosmia/hyposmia. This multifactorial symptom is associated with hyperadrenergic state and hypovolemia, among other factors that may have been present in COVID-19. Similarly, the elevated frequency of xerostomia could be associated with different causes.

Our COMPASS-31 score is almost 2-fold higher in healthy individuals than noted in previous studies, and another analysis reduced the overstatement of gastrointestinal symptoms as a bias. As the patients were asked about the presence and progression of such symptoms during COVID-19, our findings confirm these symptoms in the acute disease, especially in patients with subjective OD. Our scores are comparable to those of outpatients with post-COVID, using the same instrument. Orthostatic hypotension and hyposmia/hypogeusia occurred in 13.8 and 37.1% of them; 9 autonomic symptoms may start within the first weeks of illness, or after hospital discharge. 10 32 33

The most frequent autonomic symptoms in patients referred to autonomic testing after COVID-19 were orthostatic such as lightheadedness (93%) and headache (22%). Orthostatic intolerance without tachycardia or hypotension was more frequent than postural orthostatic tachycardia syndrome. 34 Cardiovascular reflex alterations in early COVID-19 without associated diseases were remarkable in those with mild disease or with confirmed interstitial pneumonia. Orthostatic hypotension frequency was 33%. 11

Impaired consciousness occurred in 18% of our sample. Syncope and presyncope occurred around 4.2% in COVID-19, mainly due to unexplained causes. 35 As a deficient compensatory heart rate increase was observed in a few patients with syncope during the acute hypocapnic hypoxemia, researchers hypothesize that SARS-CoV-2 could have caused ACE2 internalization in midbrain nuclei changing the baroreflex and chemoreceptor responses. 36

The neuropathogenesis in COVID-19 includes a direct attack on the nervous system, and indirect effects of systemic factors, with postinfectious immune-mediated complications. 37 The neurotropism of SARS-CoV-2 may partially contribute to respiratory failure. 38 Older adults and those with multiple diseases have reduced homeostatic capabilities. The happy or silent hypoxemia, a term applied to the deficient blood oxygenation without dyspnea, is a blood-gas disorder that may occur due to an autonomic impairment. The allostasis, coordinated alterations to maintain homeostasis, is present in COVID-19 stress response. 39

In conclusion, this study has limitations. Data collection was during the increasing rate of cases. To reduce the risk of in-hospital contamination, owing to the access of units with and without COVID-19 cases, it was not possible to recruit controls. We could not use objective methods to measure sense functions. We were unable to collect the duration of flow oxygen therapy, which excites nerve endings (common chemical sense) to cause burning sensations. They can confound taste symptoms and xerostomia. Given the small number of patients without diabetes, but with anosmia, a subcategory analysis was not possible.

The COVID-19 neurological complications are associated with a poor in-hospital outcome and a high risk of mortality. 40 However, the growing evidence of OD as a prognostic marker of better in-hospital outcome for patients with COVID-19 was not observed in this study, probably due to the sample size ( Supplementary Material ). 4

The causes underlying the neurological symptoms of inpatients with COVID-19, especially those with associated diseases, can be multiple and unclear. Our results reinforce that they can be related to the COVID-19 itself, comorbidities, pharmacotherapies, and other factors. Given that the symptoms may persist, their recognition is critical to recovery, mainly those not frequently explored. The management of orthostatic symptoms could reduce the severity of chronic illness in long-COVID.

## References

[1] Yachou Y El Idrissi A Belapasov V Ait Benali S Neuroinvasion, neurotropic, and neuroinflammatory events of SARS-CoV-2: understanding the neurological manifestations in COVID-19 patients Neurol Sci 2020 41 10 2657 2669 32725449 10.1007/s10072-020-04575-3 PMC7385206

[2] Mao L Jin H Wang M Neurologic Manifestations of Hospitalized Patients With Coronavirus Disease 2019 in Wuhan, China JAMA Neurol 2020 77 06 683 690 32275288 10.1001/jamaneurol.2020.1127 PMC7149362

[3] Iadecola C Anrather J Kamel H Effects of COVID-19 on the Nervous System Cell 2020 183 01 16 27.e1 32882182 10.1016/j.cell.2020.08.028 PMC7437501

[4] Sampaio Rocha-Filho P A Magalhães J E Fernandes Silva D Neurological manifestations as prognostic factors in COVID-19: a retrospective cohort study Acta Neurol Belg 2022 122 03 725 733 35060095 10.1007/s13760-021-01851-7 PMC8776373

[5] Amanat M Rezaei N Roozbeh M Neurological manifestations as the predictors of severity and mortality in hospitalized individuals with COVID-19: a multicenter prospective clinical study BMC Neurol 2021 21 01 116 33726699 10.1186/s12883-021-02152-5 PMC7960879

[6] Rocha-Filho P AS Magalhães J E Headache associated with COVID-19: Frequency, characteristics and association with anosmia and ageusia Cephalalgia 2020 40 13 1443 1451 33146035 10.1177/0333102420966770 PMC7645592

[7] Lechien J R Chiesa-Estomba C M De Siati D R Olfactory and gustatory dysfunctions as a clinical presentation of mild-to-moderate forms of the coronavirus disease (COVID-19): a multicenter European study Eur Arch Otorhinolaryngol 2020 277 08 2251 2261 32253535 10.1007/s00405-020-05965-1 PMC7134551

[8] Baker J Incognito A V Wilson R JA Raj S R Syncope and silent hypoxemia in COVID-19: Implications for the autonomic field Auton Neurosci 2021 235 102842 34246957 10.1016/j.autneu.2021.102842 PMC8258030

[9] Buoite Stella A Furlanis G Frezza N A Valentinotti R Ajcevic M Manganotti P Autonomic dysfunction in post-COVID patients with and witfhout neurological symptoms: a prospective multidomain observational study J Neurol 2022 269 02 587 596 34386903 10.1007/s00415-021-10735-y PMC8359764

[10] Dani M Dirksen A Taraborrelli P Autonomic dysfunction in ‘long COVID’: rationale, physiology and management strategies Clin Med (Lond) 2021 21 01 e63 e67 33243837 10.7861/clinmed.2020-0896 PMC7850225

[11] Milovanovic B Djajic V Bajic D Assessment of Autonomic Nervous System Dysfunction in the Early Phase of Infection With SARS-CoV-2 Virus Front Neurosci 2021 15 640835 34234638 10.3389/fnins.2021.640835 PMC8256172

[12] Eshak N Abdelnabi M Ball S Dysautonomia: An Overlooked Neurological Manifestation in a Critically ill COVID-19 Patient Am J Med Sci 2020 360 04 427 429 32739039 10.1016/j.amjms.2020.07.022 PMC7366085

[13] Atkins J L Masoli J AH Delgado J Preexisting Comorbidities Predicting COVID-19 and Mortality in the UK Biobank Community Cohort J Gerontol A Biol Sci Med Sci 2020 75 11 2224 2230 32687551 10.1093/gerona/glaa183 PMC7454409

[14] Vakili K Fathi M Hajiesmaeili M Neurological Symptoms, Comorbidities, and Complications of COVID-19: A Literature Review and Meta-Analysis of Observational Studies Eur Neurol 2021 84 05 307 324 34044408 10.1159/000516258 PMC8247834

[15] Larsen N W Stiles L E Miglis M G Preparing for the long-haul: Autonomic complications of COVID-19 Auton Neurosci 2021 235 102841 34265539 10.1016/j.autneu.2021.102841 PMC8254396

[16] Sletten D M Suarez G A Low P A Mandrekar J Singer W COMPASS 31: a refined and abbreviated Composite Autonomic Symptom Score Mayo Clin Proc 2012 87 12 1196 1201 23218087 10.1016/j.mayocp.2012.10.013 PMC3541923

[17] Vincent A Whipple M O Low P A Joyner M Hoskin T L Patients With Fibromyalgia Have Significant Autonomic Symptoms But Modest Autonomic Dysfunction PM R 2016 8 05 425 435 26314231 10.1016/j.pmrj.2015.08.008 PMC4766072

[18] Goldstein D S The extended autonomic system, dyshomeostasis, and COVID-19 Clin Auton Res 2020 30 04 299 315 32700055 10.1007/s10286-020-00714-0 PMC7374073

[19] Di Carlo D T Montemurro N Petrella G Siciliano G Ceravolo R Perrini P Exploring the clinical association between neurological symptoms and COVID-19 pandemic outbreak: a systematic review of current literature J Neurol 2021 268 05 1561 1569 32740766 10.1007/s00415-020-09978-y PMC7395578

[20] Soltani S Tabibzadeh A Zakeri A COVID-19 associated central nervous system manifestations, mental and neurological symptoms: a systematic review and meta-analysis Rev Neurosci 2021 32 03 351 361 33618441 10.1515/revneuro-2020-0108

[21] LEOSS Study Group Kleineberg N N Knauss S Gülke E Neurological symptoms and complications in predominantly hospitalized COVID-19 patients: Results of the European multinational Lean European Open Survey on SARS-Infected Patients (LEOSS) Eur J Neurol 2021 28 12 3925 3937 34411383 10.1111/ene.15072 PMC8444823

[22] Buscemi S Corleo D Randazzo C Risk Factors for COVID-19: Diabetes, Hypertension, and Obesity Adv Exp Med Biol 2021 1353 115 129 35137371 10.1007/978-3-030-85113-2_7

[23] Ejaz H Alsrhani A Zafar A COVID-19 and comorbidities: Deleterious impact on infected patients J Infect Public Health 2020 13 12 1833 1839 32788073 10.1016/j.jiph.2020.07.014 PMC7402107

[24] Shibata S Arima H Asayama K Hypertension and related diseases in the era of COVID-19: a report from the Japanese Society of Hypertension Task Force on COVID-19 Hypertens Res 2020 43 10 1028 1046 32737423 10.1038/s41440-020-0515-0 PMC7393334

[25] Zhao Y Liu Y Yi F Type 2 diabetes mellitus impaired nasal immunity and increased the risk of hyposmia in COVID-19 mild pneumonia patients Int Immunopharmacol 2021 93 107406 33601246 10.1016/j.intimp.2021.107406 PMC7826056

[26] Chan J YK García-Esquinas E Ko O H Tong M CF Lin S Y The Association Between Diabetes and Olfactory Function in Adults Chem Senses 2017 43 01 59 64 29126164 10.1093/chemse/bjx070

[27] Kaya K S Mazı E E Demir S T Tetik F Tuna M Turgut S Relationship between progression of type 2 diabetes mellitus and olfactory function Am J Otolaryngol 2020 41 02 102365 31806250 10.1016/j.amjoto.2019.102365

[28] Greco C Di Gennaro F D'Amato C Validation of the Composite Autonomic Symptom Score 31 (COMPASS 31) for the assessment of symptoms of autonomic neuropathy in people with diabetes Diabet Med 2017 34 06 834 838 27990686 10.1111/dme.13310

[29] Singh R Arbaz M Rai N K Joshi R Diagnostic accuracy of composite autonomic symptom scale 31 (COMPASS-31) in early detection of autonomic dysfunction in type 2 diabetes mellitus Diabetes Metab Syndr Obes 2019 12 1735 1742 31564941 10.2147/DMSO.S214085 PMC6733353

[30] Yan C H Faraji F Prajapati D P Boone C E DeConde A S Association of chemosensory dysfunction and COVID-19 in patients presenting with influenza-like symptoms Int Forum Allergy Rhinol 2020 10 07 806 813 32279441 10.1002/alr.22579 PMC7262089

[31] Rodríguez Y Rojas M Ramírez-Santana C Acosta-Ampudia Y Monsalve D M Anaya J M Autonomic symptoms following Zika virus infection Clin Auton Res 2018 28 02 211 214 29497887 10.1007/s10286-018-0515-1

[32] Goldstein D S The possible association between COVID-19 and postural tachycardia syndrome Heart Rhythm 2021 18 04 508 509 33316414 10.1016/j.hrthm.2020.12.007 PMC7729277

[33] Yong S J Long COVID or post-COVID-19 syndrome: putative pathophysiology, risk factors, and treatments Infect Dis (Lond) 2021 53 10 737 754 34024217 10.1080/23744235.2021.1924397 PMC8146298

[34] Shouman K Vanichkachorn G Cheshire W P Autonomic dysfunction following COVID-19 infection: an early experience Clin Auton Res 2021 31 03 385 394 33860871 10.1007/s10286-021-00803-8 PMC8050227

[35] de Freitas R F Torres S C Martín-Sánchez F J Carbó A V Lauria G Nunes J PL Syncope and COVID-19 disease - A systematic review Auton Neurosci 2021 235 102872 34500351 10.1016/j.autneu.2021.102872 PMC8393505

[36] Canetta C Accordino S Buscarini E Syncope at SARS-CoV-2 onset Auton Neurosci 2020 229 102734 32977101 10.1016/j.autneu.2020.102734 PMC7505046

[37] Pezzini A Padovani A Lifting the mask on neurological manifestations of COVID-19 Nat Rev Neurol 2020 16 11 636 644 32839585 10.1038/s41582-020-0398-3 PMC7444680

[38] Li Y C Bai W Z Hashikawa T The neuroinvasive potential of SARS-CoV2 may play a role in the respiratory failure of COVID-19 patients J Med Virol 2020 92 06 552 555 32104915 10.1002/jmv.25728 PMC7228394

[39] González-Duarte A Norcliffe-Kaufmann L Is ‘happy hypoxia’ in COVID-19 a disorder of autonomic interoception? A hypothesis Clin Auton Res 2020 30 04 331 333 32671502 10.1007/s10286-020-00715-z PMC7362604

[40] Frontera J A Sabadia S Lalchan R A Prospective Study of Neurologic Disorders in Hospitalized Patients With COVID-19 in New York City Neurology 2021 96 04 e575 e586 33020166 10.1212/WNL.0000000000010979 PMC7905791

