# Supplementary material for: Neurological symptoms and comorbidity profile of hospitalized patients with COVID-19
Source: Arq Neuropsiquiatr. 2023 Mar 22;81(2):146–54. doi: 10.1055/s-0043-1761433 (PMC10033191; doi:10.1055/s-0043-1761433)
Supplement: Supplementary file 1 — Supplementary Material [file 10-1055-s-0043-1761433-s220042.pdf]

SUPPLEMENTARY MATERIAL

Supplementary Table S1 In-hospital standard of care of hospitalized patients with COVID-19

		OD		
	Overall	without	with	*p*-value
	(N = 100)	(N = 56)	(N = 44)	
**Administration of medications, %**				
Intravenous antibiotics	95	96.4	93.2	0.46
Intravenous heparin	18	16.1	20.5	0.57
Systemic corticosteroids	11	16.1	4.5	0.06
Antiviral (oseltamivir)	6	1.8	11.4	**0.04**
**Highest level of supplemental O2 required, %**				0.98
None	7	7.1	6.8	
Non-invasive oxygen therapy[1]	71	71.4	70.5	
Invasive mechanical ventilation	22	21.4	22.7	
**Need for vasopressor[2],%**	22	21.4	22.7	0.87
**Venous TEE, %**	17	16.1	18.2	0.78
**ICU requirement, %**	62	64.3	59.1	0.59
**Clinical outcomes, %**				0.82
Recovery	85	85.7	84.1	
Death	15	14.3	15.9	0.82
**Hospitalization**				
Delay, days[3]	5.9 ± 4.73	5.27 ± 4.52	6.7 ± 4.92	0.13
Duration, days	17.93 ± 12.48	17.3 ± 10.76	18.73 ± 14.46	0.57

Abbreviations: O2, oxygen; TEE, thromboembolic event; ICU, intensive care unit; OD, olfactory disfunction.
Notes: Values expressed as the mean ± standard deviation or percentage (%). Bold numbers indicate statistical significance. **p*-value <0.05 from analysis of variance and the Pearson chi-squared or Fisher exact tests within group the without and with OD. Olfactory dysfunction = subjective anosmia/hyposmia. [1]Nasal cannula, venti mask and non-rebreather. [2]Due to hypovolemic, septic and cardiogenic shock. [3]Between symptom onset and admission.

 © 2023. Academia Brasileira de Neurologia. All rights reserved.
